# Voxel-based morphometry in mesial temporal lobe epilepsy surgery: an exploratory Moroccan pilot study

**DOI:** 10.3389/fnins.2026.1865438

**Published:** 2026-09-09

**Authors:** Marouane Mergaoui, Mohammed Benzagmout, Fayçal Lakhdar, Said Boujraf

**Affiliations:** Clinical Neurosciences Laboratory, Faculty of Medicine, Pharmacy and Dentistry of Fez, Sidi Mohamed Ben Abdellah University, Fez, Morocco

**Keywords:** gray matter, hippocampal sclerosis, mesial temporal lobe epilepsy, temporal lobectomy, magnetic resonance imaging, voxel-based morphometry

## Abstract

**Introduction:**

Mesial temporal lobe epilepsy (MTLE) is a common focal epilepsy syndrome that may require surgery when seizures persist despite appropriate antiseizure medications. Although widespread gray matter (GM) abnormalities have been reported in MTLE, quantitative neuroimaging has not yet been implemented in the newly established epilepsy surgery programme in Fez, Morocco. This prospective observational exploratory pilot study evaluated the feasibility of implementing a whole-brain voxel-based morphometry (VBM) workflow using the Computational Anatomy Toolbox (CAT12), Statistical Parametric Mapping (SPM12), and ResectVol, and descriptively characterized GM differences before and after unilateral temporal lobectomy.

**Methods:**

Four patients with unilateral drug-resistant MTLE and eight age- and sex-matched healthy controls were prospectively recruited. Patients underwent preoperative and postoperative magnetic resonance imaging (MRI), whereas controls underwent one research MRI examination. Right-sided patient images were left–right flipped before tissue segmentation so that the epileptogenic and operated hemisphere corresponded to the left side in all patients. Exploratory whole-brain VBM maps were generated using general linear models for the contrasts controls > presurgical patients and presurgical > postsurgical patients; age, sex, and total intracranial volume (TIV) were entered as covariates in each model (voxel-level *p* < 0.001, uncorrected; cluster extent *k* > 50 voxels). These maps were used to identify anatomical regions for *post hoc* extraction of mean GM values. Regional values were compared using the Mann–Whitney-*U*-test and Wilcoxon signed-rank test.

**Results:**

The exploratory controls > presurgical contrast showed clusters in bilateral temporal, occipital, parietal, frontal, and sensorimotor regions. The exploratory presurgical > postsurgical contrast showed clusters predominantly in the ipsilateral temporal region. *Post hoc* paired comparisons showed lower postoperative mean GM values in the four studied regions. No postoperative GM increases were observed at the exploratory mapping threshold.

**Conclusion:**

Implementing this VBM workflow was feasible within a newly established Moroccan epilepsy surgery programme. The findings are preliminary, descriptive, and hypothesis-generating; they do not establish disease mechanisms or postoperative reorganization. Larger, adequately powered longitudinal studies using dedicated repeated-measures models and multimodal quantitative MRI are required.

## 1 Introduction

Epilepsy affects approximately 1% of the global population (Liu et al., 2024). In Morocco, the estimated prevalence is 1.98% for lifetime epilepsy and 1.76% for active epilepsy (Souirti et al., 2023).

Mesial temporal lobe epilepsy (MTLE) is a common focal epilepsy syndrome in adults (Téllez-Zenteno and Hernández-Ronquillo, 2012). When seizures persist despite appropriate antiseizure medications (ASMs), drug-resistant MTLE can substantially impair quality of life and cognition and is associated with increased mortality (Engel, 2016).

MTLE is commonly associated with hippocampal sclerosis (MTLE-HS), one of its most characteristic pathological substrates, characterized by neuronal loss, gliosis, and reorganization of hippocampal circuits (Tatum, 2012; Villamizar-Torres et al., 2024). Although seizures primarily arise from mesial temporal structures, structural abnormalities may extend beyond the hippocampus. More broadly, structural and network-based studies in epilepsy have reported abnormalities involving distributed cortical, subcortical, thalamic, and hippocampal networks (Bernhardt et al., 2009; Bonilha et al., 2010; Caciagli et al., 2020; Li et al., 2017).

Voxel-based morphometry (VBM) studies have reported gray matter (GM) differences in temporal, parietal, frontal, thalamic, and occipital regions in addition to the hippocampus. These observations have motivated the hypothesis that MTLE involves distributed structural networks rather than a single isolated structure (Keller et al., 2004; Keller and Roberts, 2008; Riederer et al., 2020).

Surgical resection is an established treatment for appropriately selected patients with drug-resistant MTLE. Anterior temporal lobectomy remains a reference procedure against which newer, less invasive approaches are compared. The effectiveness of temporal lobe surgery has been established in appropriately selected patients, while subsequent studies have compared open resection with selective or minimally invasive surgical approaches (Wiebe et al., 2001; Rangwala et al., 2024; Dong et al., 2025; Ekman et al., 2024).

Technical refinements aim to improve anatomical targeting and reduce surgical morbidity, while minimally invasive procedures may also be considered in selected situations, including after unsuccessful previous temporal surgery (Fava et al., 2024; Hwang et al., 2020).

Epilepsy surgery is now performed in Fez, Morocco, broadening national access to surgical treatment (Kissani et al., 2020). This programme builds on the earlier experience of the Rabat-based team, which helped establish epilepsy surgery in Morocco (Ouazzani et al., 2013).

Conventional visual assessment may have limited sensitivity to subtle or spatially distributed structural abnormalities in MTLE-HS. Quantitative magnetic resonance imaging (MRI) methods can provide reproducible *in vivo* measures of brain morphology and complement routine clinical interpretation.

Automated structural MRI approaches have also shown potential for characterizing individual patterns of brain morphology in temporal lobe epilepsy, although the direction and distribution of the reported abnormalities remain heterogeneous across patients and studies (Focke et al., 2012; Zubal et al., 2025).

Comparable quantitative morphometric data from North African epilepsy surgery programmes remain limited. Because the present study represents an initial experience in a newly established programme and includes a small pilot cohort, an exploratory whole-brain approach was selected rather than a hypothesis-driven analysis of predefined regions.

VBM provides an automated whole-brain assessment of regional GM volume and has been widely applied in MTLE. Other quantitative MRI (qMRI) techniques, including relaxometry, diffusion imaging, and susceptibility mapping, may provide greater microstructural specificity but require additional acquisition and processing resources. VBM was therefore selected as a feasible method for establishing local quantitative neuroimaging capability using available structural MRI data.

The primary objective of this prospective observational exploratory pilot study was to assess the feasibility of implementing a whole-brain VBM workflow incorporating the Computational Anatomy Toolbox (CAT12), Statistical Parametric Mapping (SPM12), and ResectVol within a newly established epilepsy surgery programme in Morocco. The secondary objective was to descriptively characterize GM differences between presurgical patients with MTLE and healthy controls and between preoperative and postoperative MRI examinations. All analyses were considered exploratory and hypothesis-generating.

## 2 Materials and methods

### 2.1 Study design

This prospective observational exploratory pilot study with a methodological feasibility component was conducted at University Hospital Hassan II, Fez, Morocco. The study evaluated whether a whole-brain VBM workflow could be implemented using structural MRI data acquired from patients in the institutional epilepsy surgery pathway and research MRI data acquired from healthy controls. The quantitative processing workflow was performed specifically for this research study and was not part of routine clinical care.

Two comparisons were prespecified: a cross-sectional comparison of presurgical patients with MTLE and age- and sex-matched healthy controls, and an exploratory preoperative-versus-postoperative comparison within the patient cohort.

### 2.2 Participants

Between December 2023 and April 2025, four consecutive patients with unilateral drug-resistant MTLE who underwent unilateral temporal lobectomy and eight healthy volunteers were prospectively recruited. Patients were recruited from the institutional epilepsy surgery programme after multidisciplinary evaluation confirmed unilateral MTLE-HS and eligibility for temporal lobectomy. Exclusion criteria were major structural abnormalities other than mesial temporal sclerosis, bilateral or non-lesional MTLE, inability to cooperate with examinations, MRI contraindications, or intellectual disability. Postsurgical histopathological examination showed mesial temporal gliosis or sclerosis in all patients. Healthy controls were recruited from the local community, matched to the patient group by age and sex, and had no history of neurological or psychiatric disease, epilepsy, or structural abnormality on MRI.

### 2.3 MRI data collection

All MRI examinations were acquired at the Department of Radiology, University Hospital Hassan II, Fez, Morocco, using the same 1.5-T Signa HDxt scanner (GE Healthcare, Milwaukee, WI, USA) with an eight-channel neurovascular head coil. Patients underwent MRI as part of their clinical presurgical and postsurgical pathway; controls underwent MRI solely for research comparison under the approved protocol. Written informed consent covered study participation, MRI acquisition where applicable, and research use of imaging data. A three-dimensional fast spoiled gradient-echo sequence with ASSET parallel imaging was acquired using the following parameters: repetition time, 7.6 ms; echo time, 3.3 ms; inversion time, 400 ms; flip angle, 12°; slice thickness, 1.2 mm; interslice spacing, 0.6 mm; reconstructed voxel size, 0.469 × 0.469 × 0.600 mm; and 240 slices. Patients were scanned preoperatively within 3 months before surgery and postoperatively 6–18 months after surgery, after acute edema and perilesional signal changes had resolved. Controls were scanned once.

### 2.4 MRI data processing

Processing was performed in MATLAB using CAT12 (version 12; http://www.neuro.uni-jena.de/cat/) within SPM12 (Wellcome Centre for Human Neuroimaging, London, UK) and ResectVol. The workflow proceeded chronologically through reorientation and laterality harmonization, tissue segmentation, postsurgical lacuna segmentation, spatial normalization, modulation, smoothing, and quality control.

Reorientation and laterality harmonization. All T1-weighted images were manually reoriented in SPM12 before segmentation by aligning them with standard anatomical planes and setting the anterior commissure as the coordinate origin. For the two patients with right-sided MTLE, both their preoperative and postoperative T1-weighted images were left–right flipped at this stage; images from left-sided patients and all control images were not flipped. Consequently, the epileptogenic and operated hemisphere is displayed on the left in all patient maps. In the text, tables, and figures, “left” denotes the harmonized ipsilateral lesion side for patient-derived results, whereas “right” denotes the contralateral side. These labels should not be interpreted as native anatomical laterality for the two originally right-sided cases.

Tissue segmentation. Reoriented images were segmented into GM, white matter (WM), and cerebrospinal fluid (CSF) using CAT12. Segmentation used adaptive maximum a posteriori (AMAP) estimation and partial-volume estimation (PVE) at tissue interfaces.

Postsurgical lacuna segmentation. Surgical cavities were segmented from postoperative T1-weighted images using ResectVol (Casseb et al., 2021). Presurgical anatomical atlases were non-rigidly registered to each postoperative scan, and subject-specific lacuna masks were used to identify resected voxels. Voxels inside the resection cavity were excluded from GM classification; postoperative regional values therefore represent remaining GM outside the lacuna.

Spatial normalization and modulation. Segmented tissue maps were non-linearly normalized to Montreal Neurological Institute (MNI) space using the high-dimensional DARTEL-like procedure implemented in CAT12. Normalized GM maps were modulated by the Jacobian determinants of the deformation fields to preserve regional volume.

Smoothing. Modulated GM maps were smoothed with an isotropic 8-mm full width at half maximum (FWHM) Gaussian kernel.

Quality control. Reorientation and laterality were visually verified before segmentation. GM, WM, and CSF maps were inspected for incomplete coverage, misclassification, and segmentation failure. ResectVol lacuna masks were inspected before normalization. Normalized modulated GM maps were overlaid on the MNI template to assess registration, with particular attention to periresection regions. CAT12 image quality reports were reviewed for every scan. No scan was excluded because of preprocessing or quality-control failure.

### 2.5 Statistical analysis

The voxel-wise analysis was used as an exploratory mapping step rather than as a separate confirmatory inferential analysis. General linear models (GLMs) were specified in SPM12/CAT12 for the contrasts controls > presurgical patients and presurgical > postsurgical patients. Age, sex, and total intracranial volume (TIV) were entered as covariates in each voxel-wise model. The inclusion of these covariates was intended to reduce the influence of demographic and global volumetric differences on voxel-wise group comparisons, consistent with methodological considerations previously described for VBM studies (Pell et al., 2008). Clusters were displayed at a voxel-level threshold of *p* < 0.001, uncorrected, with a cluster-extent threshold of *k* > 50 voxels. Because the maps were used to localize candidate anatomical regions, corrected cluster-level inference and peak-coordinate tables were not generated. For transparency, the preoperative–postoperative voxel-wise GLM did not include a subject-specific repeated-measures factor; accordingly, it was treated as exploratory and not as a dedicated longitudinal within-subject model.

Anatomical regions showing apparent GM differences on these exploratory maps were labelled, and mean values were extracted from the smoothed, modulated, normalized GM images for *post hoc* descriptive evaluation. Cross-sectional regional comparisons between presurgical patients and controls used the Mann–Whitney *U* test. Paired preoperative-postoperative regional comparisons used the Wilcoxon signed-rank test. Analyses and visualizations were performed in Python 3.13.5.

**Figure 1:**
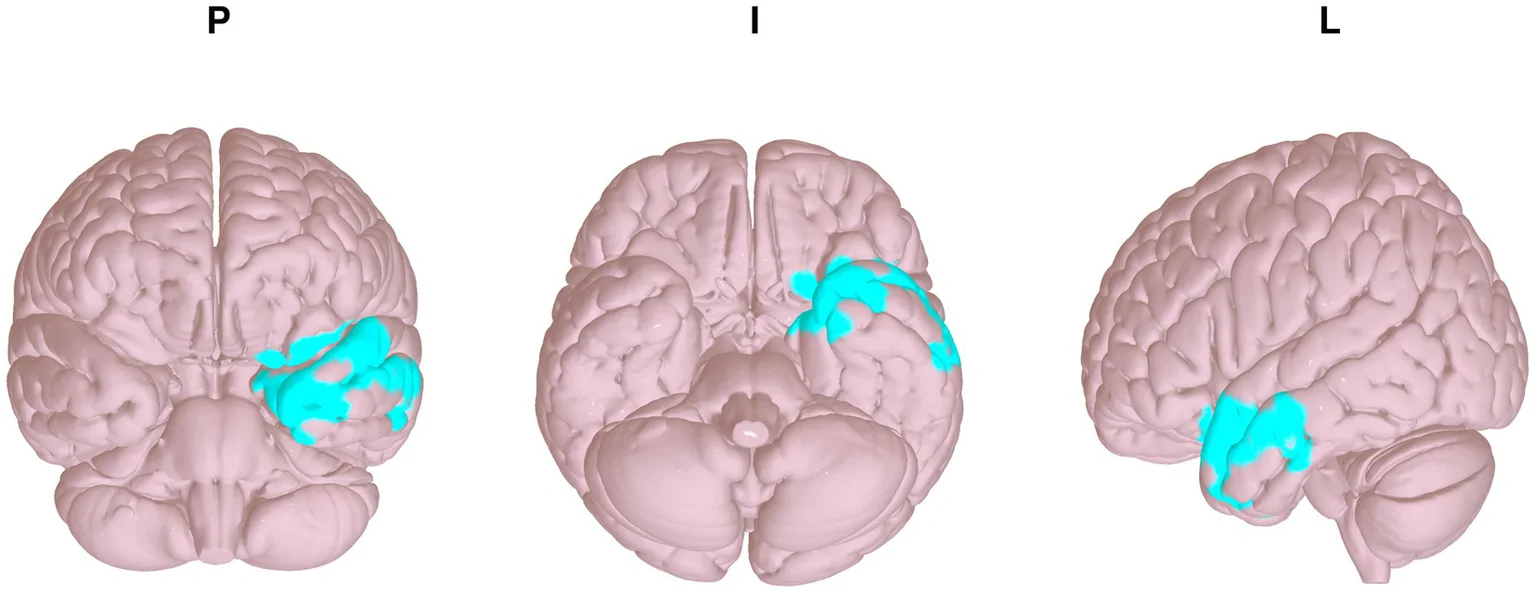
Overlap of surgical lacunae in the operated group. Lacunae were segmented from postoperative T1-weighted images using ResectVol. Images from patients with native right-sided MTLE were left–right flipped before preprocessing; therefore, the left side represents the harmonized ipsilateral operated hemisphere.

Given the pilot sample, *p*-values are reported descriptively and should not be interpreted as confirmatory evidence. Effect sizes and 95% confidence intervals are also presented to describe the magnitude and uncertainty of observed differences; all estimates remain imprecise because of the small sample (Figure 1).

## 3 Results

### 3.1 Participant characteristics

The study included four patients with MTLE and eight healthy controls (Table 1). Mean age was 30.5 years (SD 10.14) in the patient group and 32.5 years (SD 10.70) in the control group; each group was 50% female. Two patients had native left-sided lesions and two had native right-sided lesions. Patient seizure and treatment characteristics are summarized in Table 1.

**Table 1:** Clinical and demographic characteristics of patients with mesial temporal lobe epilepsy (MTLE) and healthy controls.

Variables	MTLE patients (*n* = 4)	Healthy controls (*n* = 8)
Age (y), mean (SD)	30.5 (10.14)	32.5 (10.70)
Sex, female, *n* (%)	2 (50%)	4 (50%)
Handedness	Right-handed 100%	Right-handed 100%
Seizure type, *n* (%)	SGTC: 2 (50%) FIAS: 1 (25%) FAS: 1 (25%)	
Age at seizure onset, mean (range), years	19.25 (5–35)	
Seizure frequency, *n* (%)	Daily 3 (75%) Weekly 1 (25%)	
Seizure duration, mean (range), min	8.5 (5–12)	
Epilepsy duration, mean (range), years	11.25 (4–20)	
Prodromes, *n* (%)	With 3 (75%) Without 1 (25%)	
Number of ASMs, *n* (%)	1: 1 (25%) 2: 2 (50%) 4: 1 (25%)	
Native lesion side on MRI, *n* (%)	Right: 2 (50%) Left: 2 (50%)	

ASM, antiseizure medication; FAS, focal aware seizure; FIAS, focal impaired awareness seizure; MRI, magnetic resonance imaging; MTLE, mesial temporal lobe epilepsy; SGTC, secondarily generalized tonic–clonic seizure.

### 3.2 Exploratory whole-brain VBM maps

**Figure 2:**
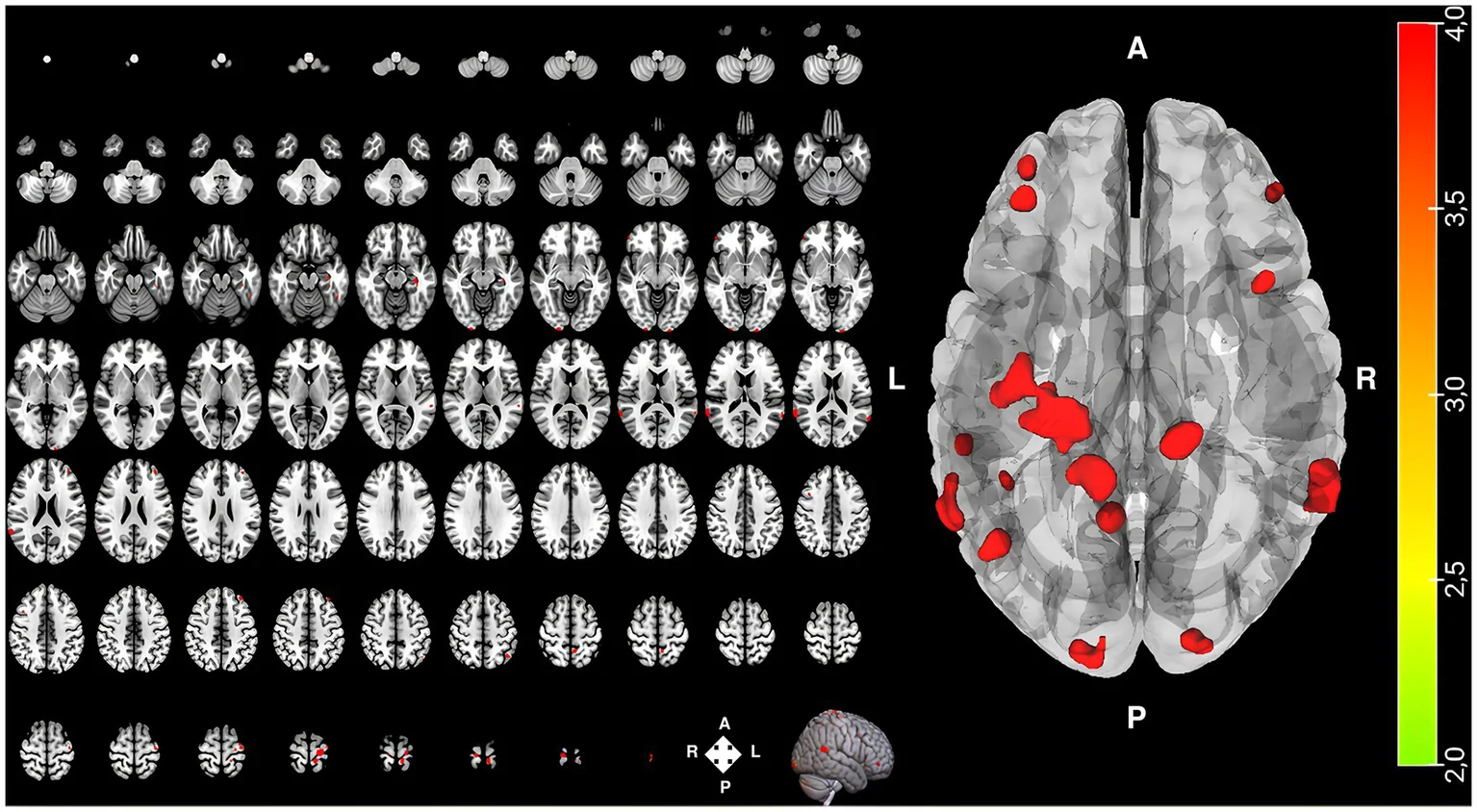
Exploratory whole-brain gray matter map for the contrast controls > presurgical patients with mesial temporal lobe epilepsy (MTLE). Maps were displayed at voxel-level *p* < 0.001, uncorrected, with cluster extent *k* > 50 voxels. Axial slices (top) show thresholded clusters on the mean brain template; the three-dimensional rendering (bottom) shows the same clusters. These maps were used to identify candidate anatomical regions for *post hoc* extraction and are not presented as corrected cluster-level inferential results. In patient-derived displays, the left side represents the harmonized ipsilateral lesion hemisphere.

For the controls > presurgical patients contrast, the exploratory whole-brain VBM map showed clusters in bilateral temporal, occipital, parietal, frontal, and sensorimotor regions (Figure 2). These thresholded clusters were used to identify candidate anatomical regions for *post hoc* extraction; they were not treated as results of a separate corrected cluster-level inferential analysis.

**Figure 3:**
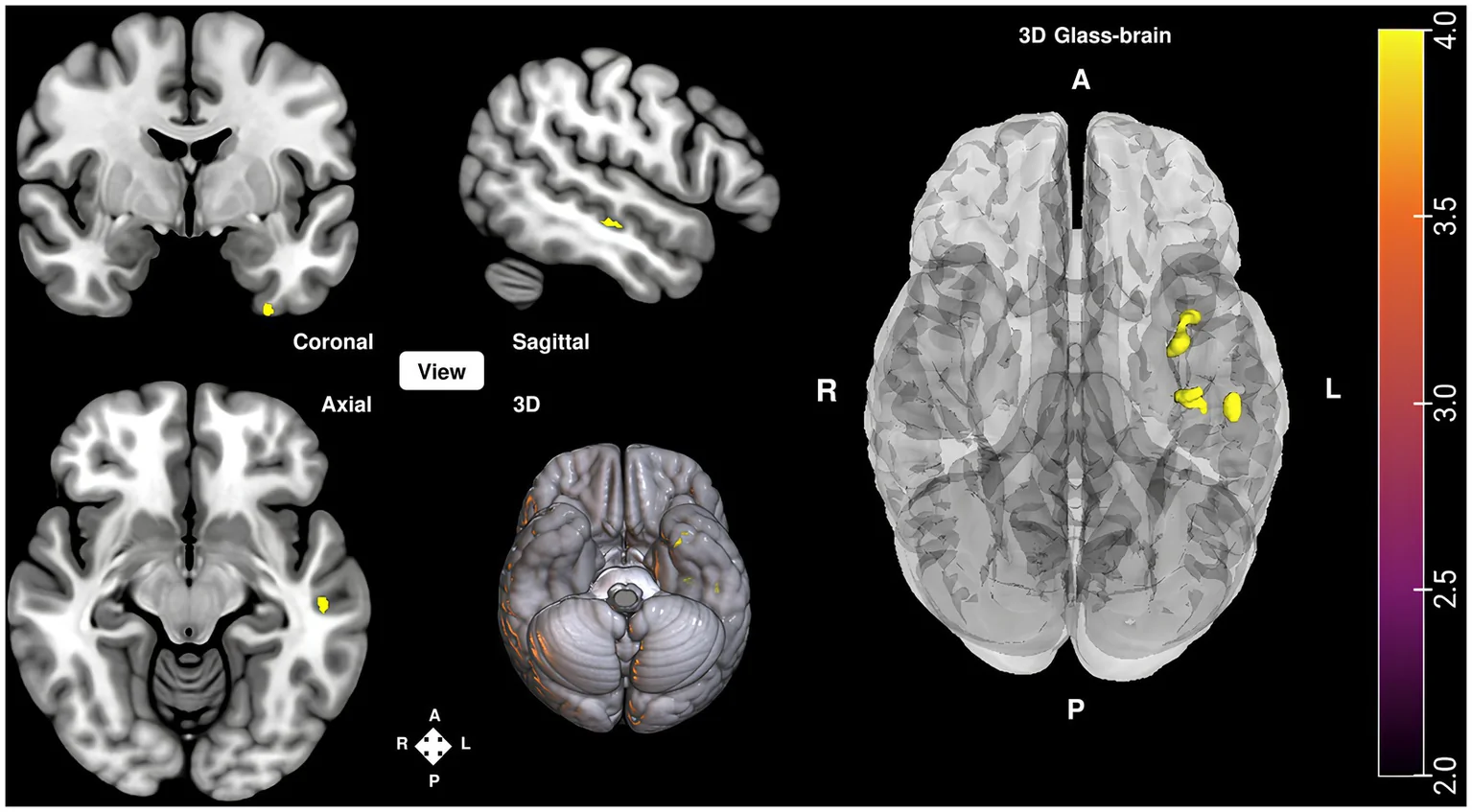
Exploratory whole-brain gray matter map for the contrast presurgical > postsurgical patients with mesial temporal lobe epilepsy (MTLE). Maps were displayed at voxel-level p < 0.001, uncorrected, with cluster extent *k* > 50 voxels. Axial slices (top), coronal slices (middle), and a three-dimensional surface rendering (right) show clusters concentrated in the harmonized ipsilateral temporal region. The color scale indicates the T statistic. No clusters were observed for the reverse contrast at this threshold. These maps are descriptive and hypothesis-generating.

For the presurgical > postsurgical contrast, the exploratory map showed clusters predominantly within the harmonized ipsilateral temporal region, including the inferior temporal, parahippocampal, fusiform, lingual, and residual hippocampal regions (Figure 3). No clusters were observed for the reverse contrast at the same exploratory threshold.

### 3.3 *Post hoc* quantitative analysis of VBM-derived anatomical regions

**Figure 4:**
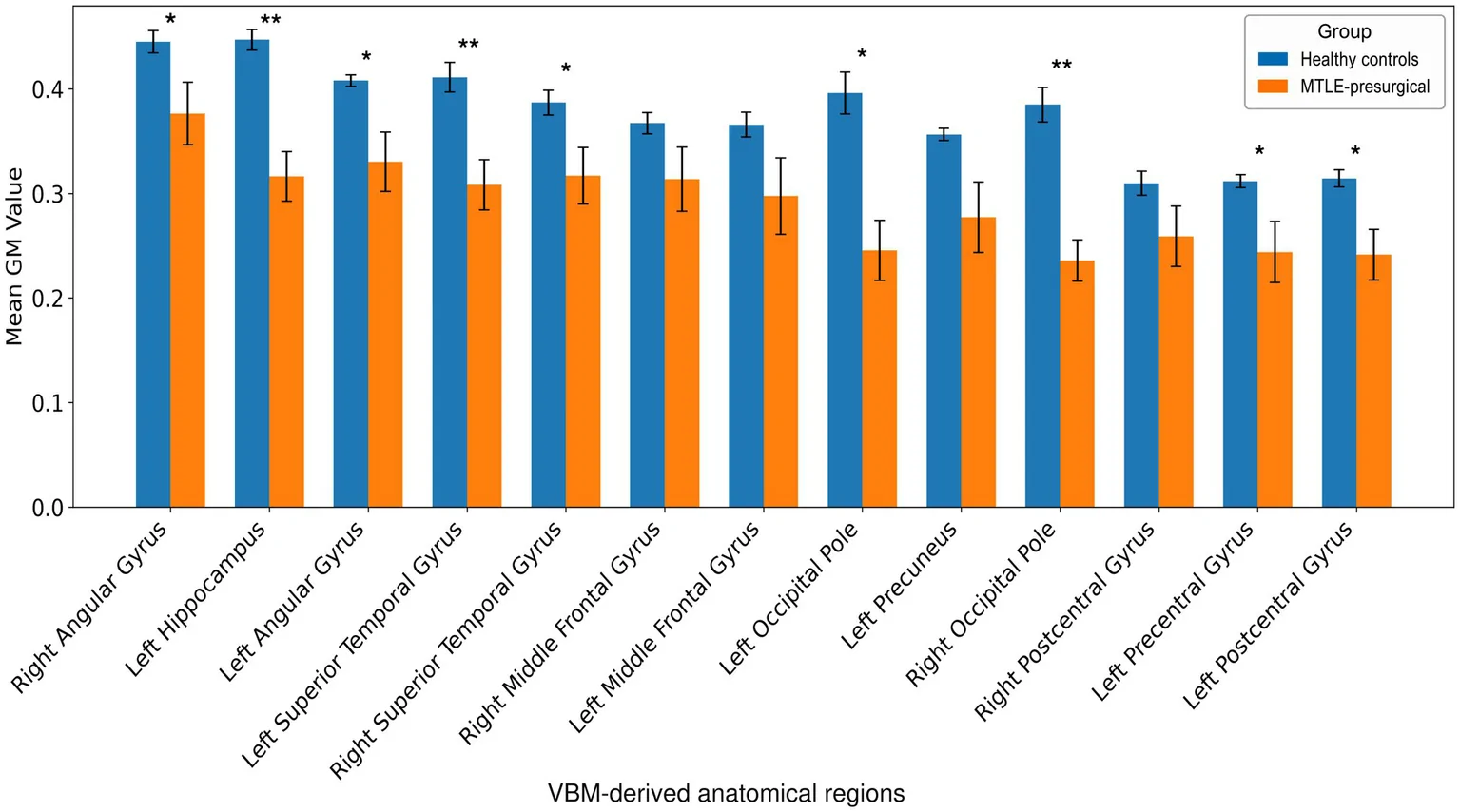
*Post hoc* mean gray matter (GM) values in anatomical regions identified on the exploratory controls > presurgical voxel-based morphometry map. Error bars show the standard error. Non-overlap of error bars was not used to infer statistical significance. Exact descriptive comparisons are reported in Table 2.

Table 2 and Figure 4 summarize mean GM values extracted from 13 anatomical regions identified on the exploratory controls > presurgical map. Mean values were lower in the presurgical group than in controls across all reported regions. The largest standardized differences were observed in the harmonized ipsilateral hippocampal region (Cohen’s *d* = −3.76, 95% CI [−5.59, −2.56]) and the bilateral occipital poles (left: *d* = −2.63, 95% CI [−3.91, −1.73]; right: *d* = −3.35, 95% CI [−5.56, −1.97]). Exact descriptive *p*-values and confidence intervals are provided in Table 2.

**Table 2:** Post hoc quantitative comparison of mean gray matter (GM) values extracted from anatomical regions identified on the exploratory controls > presurgical voxel-based morphometry (VBM) map.

Anatomical region	Presurgical MTLE GM, mean ± SD [95% CI]	Control GM, mean ± SD [95% CI]	Descriptive *p*-value	Cohen’s *d* [95% CI]
Right angular gyrus	0.38 ± 0.06 [0.28, 0.47]	0.45 ± 0.03 [0.42, 0.47]	0.05	−1.66 [−5.20, −0.07]
Left hippocampus	0.32 ± 0.05 [0.24, 0.39]	0.45 ± 0.03 [0.42, 0.47]	0.004	−3.76 [−5.59, −2.56]
Left angular gyrus	0.33 ± 0.06 [0.24, 0.42]	0.41 ± 0.02 [0.40, 0.42]	0.01	−2.30 [−7.86, −1.27]
Left superior temporal gyrus	0.31 ± 0.05 [0.23, 0.39]	0.41 ± 0.04 [0.38, 0.45]	0.004	−2.42 [−3.04, −1.75]
Right superior temporal gyrus	0.32 ± 0.05 [0.23, 0.40]	0.39 ± 0.03 [0.36, 0.42]	0.03	−1.72 [−4.09, −0.38]
Right middle frontal gyrus	0.31 ± 0.06 [0.22, 0.41]	0.37 ± 0.03 [0.34, 0.39]	0.28	−1.29 [−4.84, 0.33]
Left middle frontal gyrus	0.30 ± 0.07 [0.18, 0.41]	0.37 ± 0.03 [0.34, 0.39]	0.21	−1.39 [−4.20, 0.09]
Left occipital pole	0.25 ± 0.06 [0.15, 0.34]	0.40 ± 0.06 [0.35, 0.44]	0.004	−2.63 [−3.91, −1.73]
Left precuneus	0.28 ± 0.07 [0.17, 0.38]	0.36 ± 0.02 [0.34, 0.37]	0.11	−2.01 [−9.45, −0.40]
Right occipital pole	0.24 ± 0.04 [0.17, 0.30]	0.39 ± 0.05 [0.35, 0.42]	0.004	−3.35 [−5.56, −1.97]
Right postcentral gyrus	0.26 ± 0.06 [0.17, 0.35]	0.31 ± 0.03 [0.28, 0.34]	0.15	−1.22 [−4.65, 0.27]
Left precentral gyrus	0.24 ± 0.06 [0.15, 0.34]	0.31 ± 0.02 [0.30, 0.33]	0.01	−1.93 [−4.90, −0.95]
Left postcentral gyrus	0.24 ± 0.05 [0.17, 0.32]	0.31 ± 0.02 [0.30, 0.33]	0.03	−2.24 [−5.32, −1.03]

Between-group comparisons used the Mann–Whitney *U* test. *p*-values are descriptive and uncorrected.

Other extracted regions included the bilateral angular, superior temporal, and middle frontal gyri; the left precuneus; the right postcentral gyrus; and the left precentral and postcentral gyri. The observed group differences and their uncertainty are reported without confirmatory interpretation because the regions were selected from the same exploratory maps used to visualize group differences.

**Figure 5:**
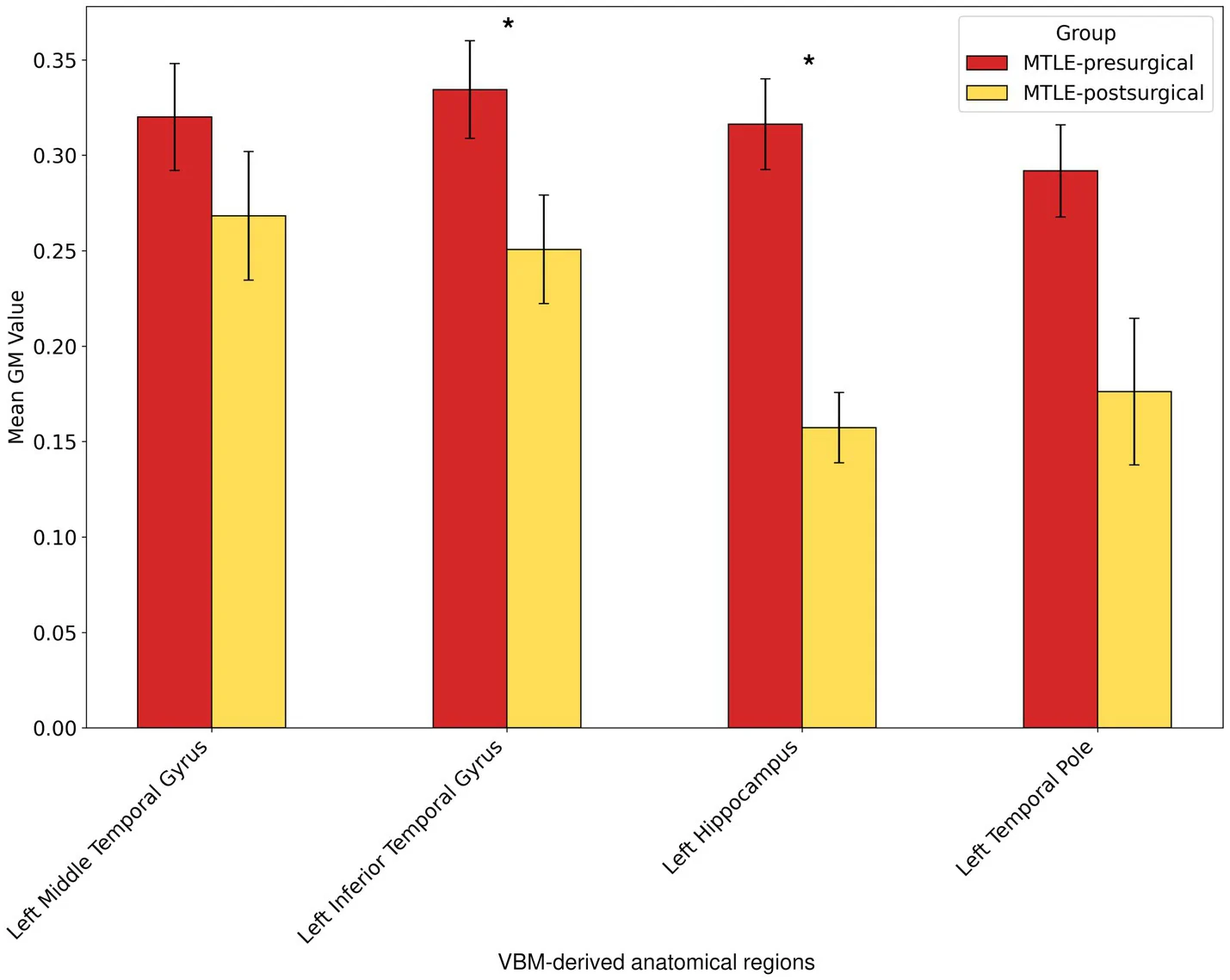
Post hoc mean gray matter (GM) values in anatomical regions identified on the exploratory presurgical > postsurgical voxel-based morphometry map. Error bars show the standard error. Exact paired descriptive comparisons are reported in Table 3.

Table 3 and Figure 5 summarize the *post hoc* paired regional comparisons. Mean postoperative GM values were lower than preoperative values in the harmonized ipsilateral middle temporal gyrus, inferior temporal gyrus, residual hippocampal region, and temporal pole. The largest standardized differences were observed in the residual hippocampal region (*d* = −9.30, 95% CI [−15.82, −2.78]) and inferior temporal gyrus (*d* = −7.53, 95% CI [−12.84, −2.22]). These estimates are highly uncertain and may reflect both biological change and residual segmentation or normalization effects near the resection cavity.

**Table 3:** Post hoc paired comparison of mean gray matter (GM) values extracted from anatomical regions identified on the exploratory presurgical > postsurgical voxel-based morphometry (VBM) map.

Anatomical region	Presurgical GM, mean ± SD [95% CI]	Postsurgical GM, mean ± SD [95% CI]	Descriptive *p*-value	Cohen’s *d* [95% CI]
Left middle temporal gyrus	0.32 ± 0.06 [0.23, 0.41]	0.27 ± 0.07 [0.16, 0.38]	0.13	−3.94 [−6.84, 1.04]
Left inferior temporal gyrus	0.33 ± 0.05 [0.25, 0.42]	0.25 ± 0.06 [0.16, 0.34]	0.04	−7.53 [−12.84, −2.22]
Left hippocampus	0.32 ± 0.05 [0.24, 0.39]	0.16 ± 0.04 [0.10, 0.22]	0.01	−9.30 [−15.82, −2.78]
Left temporal pole	0.29 ± 0.05 [0.22, 0.37]	0.18 ± 0.08 [0.05, 0.30]	0.13	−2.80 [−4.97, −0.62]

Comparisons used the Wilcoxon signed-rank test. *p*-values are descriptive and uncorrected.

## 4 Discussion

This pilot study demonstrates the practical feasibility of implementing a CAT12/SPM12/ResectVol VBM workflow in a newly established epilepsy surgery programme in Morocco. The exploratory maps and post hoc regional measurements provide preliminary observations that can inform the design of larger studies; they do not establish the mechanisms underlying MTLE or postoperative structural change.

The controls > presurgical map and subsequent regional extraction showed lower mean GM values in the harmonized ipsilateral hippocampal region and in several extra-temporal regions. Similar spatial patterns have been described in previous MTLE studies (Keller et al., 2004; Keller and Roberts, 2008; Riederer et al., 2020). In the present dataset, however, the small sample, mixed native lesion laterality, uncorrected exploratory threshold, and data-driven regional selection preclude conclusions about distributed network degeneration or region-specific functional impairment.

The presurgical > postsurgical map was concentrated in the resection territory and adjacent temporal regions. This distribution is compatible with the expected anatomical effect of temporal lobectomy, but the study cannot distinguish resected tissue from perilesional atrophy, normalization error, or other postoperative processes. The absence of clusters for the reverse contrast at the chosen threshold should likewise not be interpreted as evidence against postoperative plasticity or reorganization.

The very large standardized difference in the residual hippocampal region requires particular caution. Measurements near a surgical cavity are vulnerable to tissue misclassification and registration error, and the present study did not include native-space hippocampal subfield segmentation, diffusion MRI, or functional connectivity. Wallerian degeneration, diaschisis, and structural remodeling therefore remain hypotheses for future longitudinal multimodal studies rather than conclusions of the present analysis.

VBM measures macroscopic regional morphology and is less specific to microstructural processes than qMRI methods such as T2 relaxometry, magnetization-transfer imaging, or diffusion MRI. Diffusion-based approaches may provide complementary information about structural connectivity, including thalamocortical pathways that cannot be characterized using conventional VBM alone (Behrens et al., 2003). Surface-based morphometry may also provide complementary sensitivity to focal cortical changes. Future studies could combine these approaches with dedicated longitudinal VBM models, diffusion tractography, resting-state functional MRI, and clinical outcomes.

The present findings should therefore be interpreted primarily as evidence that the processing workflow can be implemented in this clinical setting. The study also identified practical issues that should guide the next phase, including explicit modelling of native laterality, longitudinal within-subject processing, correction for multiple comparisons, and independent validation of data-driven regions.

### 4.1 Limitations

The principal limitation is the small sample (four patients and eight controls), which provides low statistical power, imprecise effect-size estimates, and a high risk of both false-positive and false-negative findings. Additional limitations are the heterogeneous seizure characteristics and ASM regimens; mixed native lesion laterality; 1.5-T acquisition; left–right flipping, which assumes approximate hemispheric symmetry; use of an uncorrected exploratory threshold; data-driven selection of regions for *post hoc* comparisons; and the absence of seizure-outcome data. Although preoperative and postoperative regional values were compared with a paired test, the whole-brain mapping did not use a dedicated longitudinal within-subject model. Measurements near the resection cavity may combine true change with segmentation and normalization error. These limitations prevent mechanistic, functional, or clinical conclusions.

A larger longitudinal study should retain native laterality or explicitly model it, prespecify anatomical regions in an independent dataset, use dedicated repeated-measures processing, apply correction for multiple comparisons, and incorporate seizure outcomes. Covariate selection and modelling should be prespecified for each contrast. Native-space hippocampal subfield methods, diffusion MRI, and functional MRI could be added as complementary analyses.

## 5 Conclusion

This prospective observational exploratory pilot study demonstrates the feasibility of implementing a whole-brain VBM workflow incorporating CAT12, SPM12, and ResectVol within a newly established epilepsy surgery programme in Morocco. Exploratory maps identified candidate regions with apparent GM differences between presurgical patients and controls and between preoperative and postoperative examinations.

Because the cohort comprised only four patients, the uncorrected maps and *post hoc* regional comparisons are preliminary, descriptive, and hypothesis-generating. They do not establish disease mechanisms, network degeneration, functional impairment, postoperative reorganization, or surgical accuracy.

The workflow and lessons from this pilot can support larger, adequately powered longitudinal studies using dedicated repeated-measures models, correction for multiple comparisons, multimodal quantitative MRI, and clinically relevant outcomes.

## Data Availability

The raw data supporting the conclusions of this article will be made available by the authors, without undue reservation.
